# Cluster headache and galcanezumab: the first real-world Brazilian study and an expert consensus on its use among other treatments

**DOI:** 10.1186/s10194-024-01909-w

**Published:** 2024-12-03

**Authors:** Abouch Krymchantowski, Carla Jevoux, Élcio Juliato Piovesan, Marcelo Moraes Valença, Fernando Kowacs, Pedro André Kowacs, Fabíola Dach, Paulo Hélio Monzillo, Carlos Alberto Bordini, Raimundo Pereira Silva-Néto

**Affiliations:** 1 Headache Center of Rio, Rio de Janeiro, Brazil; 2 Pontifícia Universidade Católica do Rio de Janeiro (PUC-Rio), Rio de Janeiro, Brazil; 3 Hospital Municipal Miguel Couto in Rio de Janeiro, Rio de Janeiro, Brazil; 4 Universidade Federal do Paraná, Curitiba, Brazil; 5 Universidade Federal de Pernambuco, Recife, Brazil; 6 Hospital Moinhos de Vento, Porto Alegre, Brazil; 7 Instituto de Neurologia de Curitiba, Curitiba, Brazil; 8 Universidade de São Paulo, Ribeirão Preto, Brazil; 9 Hospital Israelita Albert Einstein, São Paulo, Brazil; 10 Clínica de Cefaleias de Batatais, Batatais, Brazil; 11 Universidade Federal do Delta do Parnaíba, Avenida São Sebastião, 2819, Fátima, Parnaíba, PI 64001-020 Brazil

**Keywords:** Cluster headache, Treatment, Galcanezumab, Expert consensus, Brazil

## Abstract

**Objective:**

To present the first Brazilian real-world results with galcanezumab and provide a consensus expert opinion on the prophylactic treatment of cluster headache (CH) in Brazil.

**Methods:**

The first part of the study (real-world results) was observational, prospective, uncontrolled, and descriptive. A sample of 44 consecutive patients with episodic or chronic CH were evaluated and treated in a traditional tertiary clinic from March 2020 to June 2024. The second part (consensus expert opinion) consisted of a survey completed by ten Brazilian headache clinicians with at least 25 years of clinical experience, who published at least 15 headache papers and attended at least 15 national or international headache conferences.

**Results:**

Forty-four patients (86.4% men, 13.6% women) were included. The average age was 45.9 ± 14.2 years. The diagnosis was made 27.3 ± 13.6 years after the onset of headache bouts. In 84.1% of the patients, CH was classified as episodic. Verapamil, lithium, or verapamil plus lithium were prescribed to respectively, 25%, 9.1%, and 6.8% of patients. Galcanezumab was prescribed to all and the majority (65.9%) used a dose of 300 mg once. There was a reduction in headache frequency of ≥ 50% at 3 weeks in 65.9% of patients for all doses of galcanezumab, and in 72.4% of those using galcanezumab 300 mg. Verapamil was recommended as a first-line treatment by 6 of 10 experts and a second-line treatment by the other 4 experts; galcanezumab was recommended as a first-line treatment by 4 of 10 experts and as a second-line treatment by 3 of 10 experts.

**Conclusions:**

This study presented the first real-world data with galcanezumab in Brazilian patients with CH and showed a reduction in headache frequency in most patients. A survey of Brazilian experts not meant to represent the country’s guidelines, favored galcanezumab as either the first or the second option in prophylaxis. Collectively, these results highlighted galcanezumab’s promising efficacy as a new tool in CH patients.

**Supplementary Information:**

The online version contains supplementary material available at 10.1186/s10194-024-01909-w.

## Introduction

Cluster headache (CH) is a very disabling neurological disorder, imposing a real deal of pain and desperation. It affects nearly 0.1% of the general population and typically presents with recurrent, exclusively unilateral, severe periorbital headache attacks, lasting from 15 to 180 min, and accompanied by restlessness, and ipsilateral autonomic features [1–3]. The attacks occur in bouts of varied duration, often with a predictable daily or yearly pattern. Episodic cluster headache is classified by repetitive daily attacks that last for weeks to months, followed by at least a 3-month remission period, whereas the chronic form lasts longer than one year, with remission lasting for less than 3 months [4, 5].

Current treatment strategies for CH include acute treatments, bridge or intermediate approaches, and prophylaxis. Acute treatments aim at aborting the attacks as soon as possible to impede suffering and extreme incapacitation (e.g., sumatriptan subcutaneous or oxygen inhalation). Intermediate and prophylactic treatments aim at ending the bout or exacerbation, or at least decrease the frequency and intensity of attacks. Bridge therapies are more punctual, and preventive therapies are maintained for longer periods. They are often combined in practice [6–9]. The prophylactics recommended by American and European guidelines include verapamil, lithium, topiramate, melatonin, valproic acid, and warfarin but they are not particularly effective, giving excellent relief in less than 50% of patients according to patient surveys [8–12].

Galcanezumab is a relatively newly approved treatment for CH that inhibits calcitonin gene related peptide (CGRP). Unlike verapamil, lithium, and other traditional medications whose mechanisms to treat CH are unknown, galcanezumab is a monoclonal antibody targeting the CGRP ligand, which is shown to be elevated during spontaneous CH attacks and normalized after symptomatic treatment [8–10]. Galcanezumab has been studied and has proven effective in reducing eCH, but not cCH attacks [8, 9, 13, 14]. Based on these studies, we hypothesize that galcanezumab is useful in reducing cluster headache attacks after three weeks. This study aims to present the first Brazilian results with galcanezumab for CH sufferers as well as expert opinion consensus for the approach of CH patients in Brazil.

## Methods

There are two main parts to this study: a real-world observational study of galcanezumab for cluster headache in a Brazilian clinic population, and the polling of a group of headache experts on the management of CH in Brazil to establish a clinical consensus on care. Each part is discussed separately below. Primary outcomes were reduction of attacks ≥ 50% after 3 weeks. While the secondary outcomes were to present the first Brazilian results with galcanezumab for CH sufferers and the expert opinion consensus for the approach of CH patients in Brazil.

### Real-world observational study

#### Inclusion and exclusion criteria

For sample selection, patients over 18 years diagnosed with CH according to ICHD-3 criteria [1], consecutively seen from March 2020 to June 2024, were included in this study. The study excluded pregnant women or women planning to initiate pregnancy within the next 6 months.

#### Data collection

**Fig. 1:**
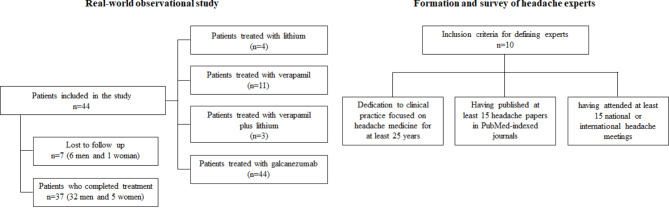
Flowchart of participants throughout the study and experts who developed the consensus

The study population consisted of patients who sought treatment at a clinic specializing in the treatment of headaches, either spontaneously or referred by other doctors, as shown in Fig. 1. After fulfilling the inclusion and exclusion criteria, patients and experts were invited to participate in the study. All patients signed the consent form. After the presentation of each case through virtual or in-person reunions, the choice of approaches and therapeutic options were discussed with at least five experts from the entire group for the real-world study. Baseline headache frequency and the time of headache history were collected by the patient’s recall information during the long-lasting initial consultations. The data regarding the cluster headache attacks after the treatment initiation were collected by headache charts filled out by the patients.

The prescription of steroids as bridge therapy, the initiation of any traditional medications, and the prescription of galcanezumab, its doses, periodicity of use, and subsequent measures were included in the studied data. Reasons for choosing one or another approach depended on previous clinical experiences and the decisions of the experts.

Patients were also asked about the adverse effects of galcanezumab at their follow-up appointments.

#### Statistical analysis

All collected data were organized in a database. The Statistical Package for Social Sciences (SPSS^®^) version 18.2.2 for statistical analysis was used. The quantitative variables were expressed as mean, standard deviation, and minimum and maximum values, while qualitative variables were expressed as absolute and relative frequencies.

### Formation and survey of headache experts

We sought to create and survey a panel of headache experts in Brazil to provide initial guidance on the use of galcanezumab relative to other CH prophylactics (Fig. 1). The inclusion criteria to define experts were: (1) dedication to clinical practice focused on headache medicine for at least 25 years; (2) having published at least 15 headache papers in PubMed-indexed journals; and (3) having attended at least 15 national or international headache meetings. Contrary to previous headache consensuses published in Brazil, this consensus was not created by a specific medical society nor depended on political issues. In addition, it was not performed to represent the country’s treatment guidelines. Three of the authors generated a survey for the expert panel that included a ranking of prophylactic treatments as well as several other questions about CH including acute treatment, imaging, and their personal experiences with CH patients.

## Results

### Real-world observational study

The studied sample consisted of 44 patients with CH, 86.4% (38/44) men and 13.6% (6/44) women. The average age was 45.9 ± 14.2 years, ranging from 18 to 78 years. Although 36.4% (*n* = 16) of the patients referred to a previous diagnosis of CH, for this study, we considered the diagnosis performed by the clinic staff, which was made 27.3 ± 13.6 years (range 2 to 55 years) after the onset of headache bouts. Every included patient had a CH history with previous bouts in the last 2 to 52 years of evolution. The frequency of the bout presentation ranged from 1 per year to 1 every 3 years. There were patients without regular intervals between bouts. Among the 7 patients with chronic presentation and a history of CH 2 to 55 years, with a mean of 24.8 years, three transited from the episodic form to the chronic form. These patients were seen an average of 1642 days after starting to present cluster headache attacks (range 365 to 4745 days). In 84.1% of the patients, CH was classified as episodic. The attacks occurred predominantly during the day (65.9%), two to three times a day (68.2%), and average duration of untreated attacks lasted between 30 and 120 min (59.1%). The mean number of attacks before and after treatment for the study population was 2.5 and 1.0, respectively. The headache features were recorded in the charts and reported by the patients. The data was analyzed, and the range was extracted (Table 1).

**Table 1:** Clinical and epidemiological characteristics of the 44 patients with cluster headache

Variables	Frequency (*n*; %; sd)
Sex	
Male	38 (86.0)
Female	6 (14.0)
Age at diagnosis (years)	
Average (SD)	45.9 ± 14.2
Interval	18–78
Latency until diagnosis (years)	
Average (SD)	27.3 ± 13.6
Interval	9–64
Patient seeking medical help	
During the first cluster headache attack and/or for the first time	5 (11.0)
From the second cluster headache attack onwards	39 (89.0)
Classification according to the interval between cluster headache periods	
Episodic cluster headache	37 (84.0)
Chronic cluster headache	7 (16.0)
Duration of the current period of cluster (days)	
Episodic cluster headache	
Average (SD)	24.2 ± 22.8
Interval	6–90
Chronic cluster headache	
Average (SD)	1,642.9 ± 1,489.0
Interval	365-4,745
Timing of attacks	
During the day	29 (66.0)
During sleep	15 (34.0)
Average duration of untreated attacks (minutes)	
< 30	14 (32.0)
30 to 120	26 (59.0)
> 120	4 (9.0)
Number of attacks/day	
Every other day	0 (0.0)
One	8 (18.2)
Two	12 (27.3)
Three	18 (40.9)
Four	6 (13.6)
Five to eight	0 (0.0)
Requesting neuroimaging exams	
During the first cluster headache attack	5 (11.4)
During the second cluster headache attack	8 (18.2)
During the third cluster headache attack	1 (2.3)
From the fourth cluster headache attack onwards	0 (0.0)

Note: SD - standard deviation

Oral steroid treatment during the initial 7–10 days was prescribed for 95.5% of patients. Initial doses ranged from 60 mg to 100 mg/ day on a tapering dose schedule. Due to similar availability and no risk of neural injuries, none of the patients received suboccipital injections of steroids. Traditional pharmacological agents, such as verapamil, lithium or verapamil plus lithium, were prescribed to 25%, 9.1%, and 6.8% of patients, respectively. Lithium was prescribed to 3 sufferers of cCH and 1 eCH patient. None received other pharmacological agents such as topiramate, melatonin, etc. Galcanezumab was prescribed to all patients and the majority (65.9%; 29/44) used a dose of 300 mg once or twice (69%) with a 30-day interval between doses. The use of steroids, galcanezumab, and traditional pharmacological agents was combined and based on the expert’s decision since it was carried out with real-world patients. Galcanezumab, as well as the other medications in Brazil, are usually purchased by the patients. Few health plans cover it. There was a reduction in headache frequency of ≥ 50% at 3 weeks in 65.9% (29/44) of patients for all doses of galcanezumab, and in 72.4% (21/29) of those using galcanezumab 300 mg. Seven patients (15.9%) to which galcanezumab was prescribed, did not return to follow-up (Table 2). Adverse effects of galcanezumab were uncommon and included local irritation (9.1%), constipation (6.8%), and upper respiratory tract infection (4.5%).

**Table 2:** Treatments used in 44 patients with cluster headache

Variables	Frequency (*n*; %)
Did they start steroid treatment?	
Yes	42 (96.0)
No	2 (4.0)
Did they start treatment with other drugs?	
Verapamil	11 (25.0)
Lithium	4 (9.0)
Verapamil plus lithium	3 (7.0)
Galcanezumab	44 (100.0)
Prescribed dose of galcanezumab	
240 mg	8 (18.0)
300 mg	29 (66.0)
360 mg	7 (16.0)
Number of times that used galcanezumab	
Once	24 (54.5)
Twice	9 (20.5)
≥ Three times	4 (9.1)
Lost to follow-up	7 (15.9)
Reduction of attacks ≥ 50% after 3 weeks	
Yes	29 (66.0)
No	8 (18.0)
Lost to follow-up	7 (16.0)

According to the treatment used for CH, there was a reduction in weekly attacks ≥ 50% after 3 weeks for all drugs in the episodic form (Table 3).

**Table 3:** Reduction of attacks ≥ 50% after 3 weeks in 44 patients with cluster headache

Form	Galcanezumab			Verapamil (episodic = 7) (chronic = 4)	Lithium (episodic = 2) (chronic = 2)
	240 mg (episodic = 8) (chronic = 0)	300 mg (episodic = 27) (chronic = 2)	360 mg (episodic = 2) (chronic = 5)		
Episodic (n; %)	4 (50.0)	20 (74.0)	2 (100.0)	5 (71.0)	2 (100.0)
Chronic n; %)	0 (0.0)	1 (50.0)	2 (40.0)	2 (50.0)	1 (50.0)

### Formations and survey of headache experts (table 4)

**Table 4:** Opinions of Brazilian experts on the treatment of cluster headaches

Questions	Expert 1	Expert 2	Expert 3	Expert 4	Expert 5	Expert 6	Expert 7	Expert 8	Expert 9	Expert 10
	Yes/No	Yes/No	Yes/No	Yes/No	Yes/No	Yes/No	Yes/No	Yes/No	Yes/No	Yes/No
Do you routinely request imaging studies in the first bout?	Yes	Yes	Yes	Yes	Yes	Yes	Yes	Yes	Yes	Yes
Even with a typical clinical picture?	Yes	Yes	Yes	Yes	No	Yes	Yes	Yes	Yes	Yes
Do you request imaging for a patient with previous bouts, without previous imaging, but with you for the first time?	No	No	No	No	No	Yes	Yes	No	Yes	No
Patient with previous bouts seen by you, with a new bout with atypical features. Do you request imaging?	Yes	Yes	Yes	No	No	No	Yes	Yes	Yes	No
Do you use bridge therapy routinely? Point the first and second options	Yes	Yes	Yes	Yes	No	Yes	Yes	Yes	Yes	Yes
steroid	1st	1st	1st	1st	1st	1st	1st	1st	1st	1st
blockade Greater Occipital Nerve, supraorbital or following the pain	2nd	2nd	2nd	2nd	2nd	No	2nd	No	2nd	No
Do you routinely combine bridge, prevention and acute treatments?	Yes	Yes	Yes	Yes	Yes	Yes	Yes	Yes	Yes	Yes
Do you use long-acting triptans twice a day routinely?	No	No	No	No	No	No	Yes	No	Yes	No
What is your acute treatment in order of preference?										
injectable sumatriptan as needed	1st	1st	1st	1st	1st	1st	1st	1st	1st	1st
nasal sumatriptan	No	No	No	No	No	No	No	No	3rd	No
other triptans	No	No	No	No	No	No	No	No	No	No
100% Oxygen	2nd	2nd	2nd	2nd	2nd	2nd	2nd	2nd	2nd	2nd
NSAID, narcotics or others	No	No	No	No	No	No	No	No	No	No
Do you combine acute treatments?	Yes	Yes	Yes	No	Yes	Yes	No	Yes	Yes	Yes
Limitations or as needed regardless of number of attacks?	limitations	limitations	limitations	limitations	limitations	as needed	limitations	limitations	limitations	as needed
Questions	Expert 1	Expert 2	Expert 3	Expert 4	Expert 5	Expert 6	Expert 7	Expert 8	Expert 9	Expert 10
	Yes/No	Yes/No	Yes/No	Yes/No	Yes/No	Yes/No	Yes/No	Yes/No	Yes/No	Yes/No
What is your preventive treatment in order of preference?										
Verapamil	2nd	2nd	2nd	1st	1st	1st	1st	1st	1st	2nd
Lithium	3rd	3rd	3rd	3rd	3rd	3rd	3rd	2nd	Don`t use	3rd
Divalproate	7th	7th	7th	7th	7th	7th	2nd	4th	Don`t use	Don`t use
Topiramate	5th	5th	5th	5th	5th	5th	4th	Don`t use	Don`t use	Don`t use
Combinations	4th	4th	4th	4th	4th	4th	5th	3rd	Don`t use	4th
onabotuliumtoxinA	Don`t use	Don`t use	Don`t use	Don`t use	Don`t use	Don`t use	8th	Don`t use	Don`t use	Don`t use
galcanezumab	1st	1st	1st	2nd	2nd	2nd	6th	Don`t use	Don`t use	1st
Melatonin	6th	6th	6th	6th	6th	6th	7th	Don`t use	Don`t use	Don`t use
Others	Don`t use	Don`t use	Don`t use	Don`t use	Don`t use	Don`t use	Don`t use	Don`t use	Don`t use	Don`t use
After how long should he/she return? (weeks)	3	5	4	2	3	4	2	2	2	4
How many patients have already been evaluated and treated?	230	197	97	100	20	100	30	84	30	98
What is the percentage of men?	85	84	81	80	80	82	90	81	70	80
What is the average age of men (years)?	32	36	34	33	38	39	30	51	30	36
What is the percentage of women?	15	16	19	20	20	18	10	19	30	20
What is the average age of women (years)?	32	31	29	27	33	39	25	38	32	32
How many with chronic cluster headache?	5	6	2	2	0	4	5	1	0	2
Treatment mostly used for chronic	V/L	V/L	V/L	V/L	V	V/L	V/L	V/L	V	V
Did you refer patients to procedures?	Yes	Yes	Yes	Yes	Yes	Yes	Yes	No	Yes	No
What is the percentage of referrals?	2.0	1.0	0.8	1.0	0.5	2.0	10.0	0.0	< 10.0	0.0
How many patients < 18 years old	3	1	0	0	0	3	2	0	0	1
Drugs used for this population	I/V/L	I	Not available	Not available	Not available	steroid	V	No	No	V

Note: V: verapamil; L: lithium; I: Indomethacin

Ten headache experts met the inclusion criteria for the survey. They were distributed throughout all regions of the country. All of them were asked 10 questions about the management of cluster headache. The answer option was “yes” or “no”.

## Discussion

The management of CH, one of the most painful and disabling forms of headache, has shifted with the development of new medications like galcanezumab, even as a first line of treatment [6, 13–15]. To the best of our knowledge, this is the first report with galcanezumab in Brazilian CH patients showing the effectiveness, tolerability, and pragmatic approach position from headache specialists for this primary headache.

### Efficacy of galcanezumab in cluster headache

The outcomes of the present study bring to light a significant decrease in the number of headache attacks, with 64.8% of the patients with eCH achieving a ≥ 50% decrease in the number of attacks within three weeks of its subcutaneous administration. They are consistent with prior research [13, 14] for the preventive treatment of eCH. Although we do not know whether some patients improved due to a spontaneous remission, our timeframe to evaluate headache frequency reduction was, as previously reported in studies [14], three weeks. However, it is noteworthy that chronic sufferers also revealed at least some degree of reduction in headache severity (42.8% of the cCH patients and 50% of those cCH who were not lost to follow up). These results are even more important since the typical first-line medication verapamil may require an uncomfortable posology (three times a day), and promote tolerability issues such as constipation and AV conduction abnormalities [4, 5].

In our study, 66% of patients had a reduction in headache attacks ≥ 50% after 3 weeks with 300 mg doses of galcanezumab. Other studies that also evaluated the efficacy of galcanezumab in eCH in real-world patients found similar results, but used a dose of 240 mg galcanezumab [16–18].

Galcanezumab is a monoclonal antibody that targets CGRP and has been shown to be an effective treatment in stopping CH attacks. CGRP is found throughout the trigeminovascular system and is elevated in the jugular blood and tear fluid of patients with CH, both interictally and during attacks [19]. There is no doubt that CGRP plays an important role in the pathophysiology of CH, as CGRP infusion induces headache attacks in CH patients by promoting activation of the trigeminal-autonomic reflex [20] and its reduction with symptomatic treatment produces improvement [8–10].

### Expert preferences and future directions

The panel of experts formed within the framework of this study also points out the need to tailor treatment for CH depending on whether CH is manifesting for the first time, the frequency and severity of the attacks, the patient’s particular conditions as the current time of bout duration and response to the previous therapy. It also describes the priority order for choosing treatment options, the likelihood of combining approaches, the place for new therapeutic agents, such as galcanezumab, and the consulting profile for the follow-up visits. Interestingly, for the whole panel of experts, galcanezumab was a treatment option to start even for chronic patients, despite its demonstrated lack of efficacy [13]. It also emphasized that non-cluster headache treatments, such as onabotulinumtoxinA, did not yet grab Brazilian paradigms of treatment for CH, despite existing questionable evidence [21].

The next steps in the assessment of galcanezumab should aim at developing more studies, especially for the heretofore understudied populations such as younger, older, and patients with cCH. Moreover, the development of further studies on the efficacy of combining galcanezumab with other traditional or novel treatments such as neuromodulation will be useful and are warranted in improving CH management.

Based on the data from the 10 Brazilian experts on the treatment of cluster headaches, which was not meant to represent the country guidelines, bring forward the following expert preferences consensus (Table 5):

**Table 5:** Expert consensus on the treatment of cluster headaches

Question consensus response	
Primary statement	
Preventive treatment preference	1st: Verapamil (7 out of 10 experts) 2nd: Galcanezumab (4 out of 10 experts consider it first-line or second-line) Lithium is commonly used as a 3rd -line treatment
Secondary statement	
Routine imaging studies in the first bout	Yes. All experts unanimously recommend imaging studies during the first bout
Imaging studies with a typical clinical picture	Yes. 9 out of 10 experts recommend imaging studies, even with a typical clinical picture
Imaging for previous bouts without prior imaging, but first time with the expert	Mixed. 6 out of 10 experts do not recommend imaging in such cases. However, 4 experts consider it necessary
Imaging for new bouts with atypical features	Yes. 7 out of 10 experts recommend imaging when new bouts present with atypical features
Routine use of bridge therapy	Yes. 9 out of 10 experts routinely use bridge therapy, primarily with steroids
First and second options for bridge therapy	1st: Steroids (unanimous). 2nd: Greater Occipital Nerve (GON) blockade or supraorbital blockade (7 out of 10 experts)
Combining bridge prevention and acute treatments	Yes. All experts combine bridge prevention and acute treatments routinely
Routine use of long-acting triptans twice a day	No. Most experts do not routinely use long-acting triptans twice a day
Order of preference for acute treatments	1st: injectable sumatriptan (unanimous). 2nd: 100% oxygen (unanimous). Nasal sumatriptan is only considered a 3rd option by one expert
Combining acute treatments	Yes. 8 out of 10 experts combine acute treatments, with limitations regarding the number of attacks
OnabotulinumtoxinA (Botox) use	No. All of experts do not use onabotulinumtoxinA for CH
Return visit interval	The return visit interval varies, with a range from 2 to 5 weeks. The most common intervals are 2 weeks (4 experts) and 4 weeks (3 experts)
Number of patients treated	The number of patients treated by each expert varies widely, with the most experienced having treated over 230 patients and the least experienced having treated around 20
Percentage of male patients	The consensus male/female ratio is approximately 4:1
Percentage of chronic cluster headaches	Chronic cluster headaches are relatively rare, with most experts reporting a prevalence of less than 5%
Preferred treatment for chronic cluster headaches	Combination of Verapamil and Lithium (V/L)
Referrals to procedures	Yes. Most experts (8 out of 10) refer patients to procedures such as nerve blocks when necessary
Treating patients under 18 years old	Rare. Few experts have experience treating patients under 18 years old, and Indomethacin or Verapamil is the preferred treatment when required

### Limitations and strengths of the study

The current study offers accountabilities of real-world experience of CH using galcanezumab with the following limitations. First, a limitation is the uncontrolled design, although the patients were consecutive. Second, bias in treatment selection was limited by the discussion of each subject between 5 experts to decide the evaluation and treatment. Third, we had a 15.9% attrition rate, which may have been related to drug effectiveness, adverse effects, or cost issues since Brazilians rarely receive it from insurance or medical plans and have to purchase the prescribe therapy [15, 21]. Thus, the study may overestimate effectiveness or underestimate adverse effects.

Another potential methodological weakness is the small number of participants despite the four-year study. The fact that the study was conducted only in one country, thus limiting the scope of results, was on purpose and may reflect the reality of a specific geographic region. Additionally, the results of this real-world observational study may not have reflected all of the preferences of the panel of experts.

Altogether, this paper provides additional information on the effectiveness and feasibility of galcanezumab in CH; the findings emphasize the use of this mAb as an effective agent in managing CH, especially eCH. The development of galcanezumab as part of a multidimensional treatment plan endorsed by consensus-based guidelines is a major step forward in the care of this highly morbid disorder. However, the findings discussed also reveal the need to keep researching more about the possibilities of long-term therapy to clarify adherence issues and tolerability as well as the real need for combining treatments.

## Conclusions

This study presented the first real-world data with galcanezumab in Brazilian patients with CH and showed a reduction in headache frequency in most patients. A survey of Brazilian experts, even not representing the country’s guidelines, favored galcanezumab as either the first or second option in prophylaxis. Collectively, these results highlighted galcanezumab’s promising efficacy as a new tool in CH patients.

## Electronic supplementary material

Below is the link to the electronic supplementary material.

Supplementary Material 1

## Data Availability

No datasets were generated or analysed during the current study.
